# Phylogenesis and Biological Characterization of a New Glucose Transporter in the Chicken (*Gallus gallus*), GLUT12

**DOI:** 10.1371/journal.pone.0139517

**Published:** 2015-10-02

**Authors:** Edouard Coudert, Géraldine Pascal, Joëlle Dupont, Jean Simon, Estelle Cailleau-Audouin, Sabine Crochet, Michel Jacques Duclos, Sophie Tesseraud, Sonia Métayer-Coustard

**Affiliations:** 1 UR83 Recherches Avicoles, Institut National de la Recherche Agronomique, Nouzilly, France; 2 Bioinformatique—Genomique Comparative et Evolutive, Institut National de la Recherche Agronomique, Castanet Tolosan, France; 3 UMR 7247 INRA-CNRS-Université de Tours-Haras Nationaux, Institut National de la Recherche Agronomique, Nouzilly, France; Tohoku University, JAPAN

## Abstract

In mammals, insulin-sensitive GLUTs, including GLUT4, are recruited to the plasma membrane of adipose and muscle tissues in response to insulin. The GLUT4 gene is absent from the chicken genome, and no functional insulin-sensitive GLUTs have been characterized in chicken tissues to date. A nucleotide sequence is predicted to encode a chicken GLUT12 ortholog and, interestingly, GLUT12 has been described to act as an insulin-sensitive GLUT in mammals. It encodes a 596 amino acid protein exhibiting 71% identity with human GLUT12. First, we present the results of a phylogenetic study showing the stability of this gene during evolution of vertebrates. Second, tissue distribution of chicken *SLC2A12* mRNA was characterized by RT-PCR. It was predominantly expressed in skeletal muscle and heart. Protein distribution was analysed by Western blotting using an anti-human GLUT12 antibody directed against a highly conserved region (87% of identity). An immuno-reactive band of the expected size (75kDa) was detected in the same tissues. Third a physiological characterization was performed: *SLC2A12* mRNA levels were significantly lowered in fed chickens subjected to insulin immuno-neutralization. Finally, recruitment of immuno-reactive GLUT12 to the muscle plasma membrane was increased following 1h of intraperitoneal insulin administration (compared to a control fasted state). Thus insulin administration elicited membrane GLUT12 recruitment. In conclusion, these results suggest that the facilitative glucose transporter protein GLUT12 could act in chicken muscle as an insulin-sensitive transporter that is qualitatively similar to GLUT4 in mammals.

## Introduction

In mammals, facilitated transport of glucose into cells is mediated by a family of facilitative glucose transporter (GLUT) proteins. Fourteen isoforms have been described in the human to date: the 12 facilitative glucose transporters GLUT1-12, HMIT (H^+^coupled myo-inositol transporter or GLUT13) and GLUT14. All of them present common sugar transporter features: 12 membrane-spanning helices, a N-linked glycosylation site and intracellular NH_2_ and COOH termini [1–2]. Based on primary sequence comparisons, the GLUT family is divided into 3 classes: Class I (GLUT1-4 and GLUT14); Class II (GLUT5, 7, 9 and 11) and Class III (GLUT6, 8, 10, 12 and HMIT). GLUT proteins have specific roles in whole body glucose homeostasis because of their substrate specificity, tissue distribution, cellular location and regulation mechanisms.

Also in mammals, some GLUTs, called insulin-sensitive GLUTs, are acutely recruited to the plasma membrane in response to insulin. GLUT4, which is expressed in insulin-sensitive tissues (i.e. skeletal muscle, the heart, adipose tissue), is the major member of this family and one of the most intensively studied glucose transporters [3] because it is responsible for the insulin-mediated increase in glucose uptake that occurs in response to elevated plasma glucose and insulin levels in the post-prandial state. Recent findings suggest that, in addition to GLUT4, GLUT12 might also contribute to insulin-stimulated glucose uptake in skeletal muscle and adipose tissue [4–7]. Indeed, insulin has been reported to stimulate the translocation of GLUT12 from intracellular membrane compartments to the plasma membrane in different models (e.g. MCF-7 breast cancer cells [4], human skeletal muscle [5]). Moreover, transgenic overexpression of this protein enhances insulin sensitivity in mice [6]. These studies suggest that GLUT12 may be a second insulin-sensitive glucose-transporter.

Birds and especially chickens exhibit particular features for glucose metabolism. For example, despite the presence of insulin circulating at “normal” concentrations, chickens present a high level of glycemia (2 g/l), and a low sensitivity to exogenous [8–10]. High doses of exogenous insulin are required to induce hypoglycemia and chickens resist huge doses of exogenous insulin, which are lethal in mammals [11]. However, chickens and ducks are not totally insensitive to exogenous insulin, which enhances the uptake of glucose in several skeletal muscles [12–13]. Moreover, immuno-neutralization of insulin in young chickens rapidly induces considerable increases in plasma levels of glucose [14]. Insulin induces a rapid although modest increase in glucose uptake by chicken myotubes, an uptake inhibited by phloretin, an inhibitor of glucose transporters [15]. Inhibitory effect of phloretin on glucose uptake has been also described in isolated muscles from English sparrows *(Passer domesticus*) [16]. These findings support the existence of functional glucose transporters in avian muscle. Nevertheless, the mechanism of control of plasma glucose in chickens remains to be elucidated as immuno-reactive GLUT1 but not GLUT4 has been detected in chicken tissues [15].

The chicken genome database contains several sequences that have been suggested to encode glucose transporter-like proteins, but none encoding a GLUT4 ortholog. In chickens, only GLUT 1, -2, -3, -8 and -9 have been partially described and characterized [17–20]. One of the nucleotide sequences is annotated as encoding a chicken GLUT12 ortholog (*SLC2A12* gene; ENSGALG00000013980). Though not yet described or characterized in chickens, it might act as an insulin-sensitive transporter in this species, similarly to GLUT4 in mammals. Phylogeny and synteny analyses were first used to confirm the lack of GLUT4 in chickens and secondly to demonstrate the existence of a *SLC2A12* gene and its stability within vertebrates during evolution. To further characterize GLUT12 in chickens, we analysed its tissue distribution and finally evaluated its potential sensitivity to insulin at mRNA and protein levels.

## Materials and Methods

### Phylogenetic and syntenic analyses

All predicted and annoted members of the GLUT family in *Gallus gallus* were obtained from the Ensembl (http://www.ensembl.org/index.html) database (Ensembl release 75). Protein IDs and chromosome location are summarized in Table 1. Analysis of avian GLUTs was performed on the Phylogeny.fr platform (http://www.phylogeny.fr [21, 22]) using Ensembl protein IDs. Sequences were aligned with MUSCLE (v3.7) configured for highest accuracy (MUSCLE with default settings). After alignment, ambiguous regions (i.e. containing gaps and/or poorly aligned) were removed with Gblocks (v0.91b) using the following parameters: minimum length of a block after gap cleaning equal to 10; no gap positions allowed in the final alignment; rejection of all segments with contiguous non-conserved positions larger than 8; minimum number of sequences for a flank position equal to 85%. The phylogenetic tree was reconstructed using the maximum likelihood method implemented in the PhyML program (v3.0 aLRT). The default substitution model was selected. Reliability of internal branches was assessed using the aLRT test (SH-Like). Graphical representation and edition of the phylogenetic tree were performed with TreeDyn (v198.3).

**Table 1:** Predicted and annoted GLUTs from chicken (*Gallus gallus*) Ensembl database.

Protein	Protein ID (Ensembl)	Chromosome location
**GLUT1**	ENSGALP00000007795	21
**GLUT2**	ENSGALP00000015129	9
**GLUT3**	ENSGALP00000036432	1
**GLUT5**	ENSGALP00000003884	21
**GLUT6**	ENSGALP00000004710	17
**GLUT8**	ENSGALP00000001249	17
**GLUT9**	ENSGALP00000031420	4
**GLUT10**	ENSGALP00000007155	20
**GLUT11**	ENSGALP00000009743	15
**GLUT12**	ENSGALP00000022613	3

Chicken GLUT12 was compared with GLUT12s from other species contained in the Ensembl database. Accession numbers are summarized in Table 2. Analyses of the orthologous relationships between different species were carried out by combining the phylogenetic reconstruction of the gene family with the syntenic comparison. The GLUT12 proteins identified were then analysed by the PhyleasProg web server (Phylogenetic Analysis Programs v2.7 [23]) with fine computation and results on orthologs and paralogs. The phylogenetic tree of the *SLC2A12* gene was displayed by Archaeopteryx. Selection pressures were acted using the PALM method of PhyleasProg [24] on the avian GLUT12 sequence. Gene arrangement of the genomic region encompassing GLUT12 was performed with the genome browser Genomicus v79.01 –PhyloView (http://www.genomicus.biologie.ens.fr) [25].

**Table 2:** Predicted and annotated GLUT12s from various species in the Ensembl database.

Species	Protein ID	Amino acid number
**Wallaby** (*Macropus eugenii)*	ENSMEUP00000005529	614
**Opossum** (*Monodelphis domestica)*	ENSMODP00000021849	627
**Mouse (** *Mus musculus)*	ENSMUSP00000043962	622
**Rat (** *Rattus norvegicus)*	ENSRNOP00000015566	621
**Rabbit (** *Oryctolagus cuniculus)*	ENSOCUP00000006133	610
**Tarsier (** *Tarsius syrichta)*	ENSTSYP00000011636	435
**Orangutan (** *Pongo pygmaeus)*	ENSPPYP00000019063	576
**Human (** *Homo sapiens)*	ENSP00000275230	617
**Macaque (** *Macaca mulatta)*	ENSMMUP00000030186	579
**Pig (** *Sus scrofa)*	ENSSSCP00000004506	621
**Cow (** *Bos taurus)*	ENSBTAP00000025291	621
**Turkey (** *Meleagris gallopavo)*	ENSMGAP00000013845	560
**Chicken (** *Gallus gallus)*	ENSGALP00000022613	596
**Zebra finch (** *Taeniopygia guttata)*	ENSTGUP00000011894	558
**Anole lizard (** *Anolis carolinensis)*	ENSACAP00000010115	552
**Frog (** *Xenopus tropicalis)*	ENSXETP00000000236	527
**Zebrafish (** *Danio rerio)*	ENSDARP00000053530	610
**Fugu (** *Takifugu rubripes)*	ENSTRUP00000007853	529
**Tetraodon (** *Tetraodon nigroviridis)*	ENSTNIP00000019417	522

### Chemicals

DNAse I was purchased from Roche Applied Science (Meylan, France).The pre-made polyacrylamide solution was obtained from Euromedex (Souffelweyersheim, France). Nitrocellulose membrane and protein standards were purchased from Bio-Rad Laboratories (Hercules, CA, USA). Odyssey blocking buffer was purchased from Li-cor (LI-COR Inc. Biotechnology, Lincoln, NE). The Membrane Protein extraction kit was purchased from PromoKine (PromoCell, Heidelberg, GER).

An anti-human GLUT12 antibody was purchased from Abcam (ab100993, Cambridge, UK). This rabbit polyclonal antibody is directed against residues 250–350 of Human GLUT12. Identity between human and predicted chicken sequences in this region is high (87%). The monoclonal anti-human vinculin antibody was from Sigma (V9131, Sigma Chemical Company). Secondary antibodies (anti-rabbit and anti-mouse IgG (H+L)) were conjugated to DyLight® 680 fluorescent dye and purchased from Cell signaling (#5366 and #5470, Cell signaling-Ozyme, Saint Quentin Yvelines, FR).

### Animals

This study was conducted in strict accordance with the European Union Guidelines for animal care. All investigators were certified by the French government to carry out animal experiments and this study was conducted under authorization 37–085 delivered to J. Dupont by the French Ministry of Agriculture. Tissues were issued from our earlier study [14], where experimental conditions are described in detail. All procedures were approved by the French Agricultural Agency and the Scientific Research Agency and conducted in accordance with the guidelines for Care and Use of Agricultural Animals in Agricultural Research and Teaching. Panels of tissues were issued from chickens reared conventionally in an environmentally controlled poultry house. All the chickens were sacrificed by decapitation and different tissues were removed, quickly frozen and stored at -80°C. All efforts were made to minimize suffering.

### Expression of SLC2A12 mRNA and protein distribution of GLUT12 in chicken tissues

At 6 weeks of age, male broiler chickens (N = 6) were slaughtered and different skeletal muscles as well as several tissues such as brain, liver, adipose tissue and heart were removed and snap frozen in liquid nitrogen.

Ontogenesis study was restricted to the *Pectoralis major* and the leg muscles, collected at 19 days during embryogenesis, hatch and 5 days post-hatch. Muscles were removed and snap frozen in liquid nitrogen.

### Insulin immune-neutralization model

Male broiler chickens were housed in an environment-controlled room and they were fed ad libitum with a conventional balanced diet based on corn, wheat, peas, soy bean, meal, corn gluten and rapeseed oil [14]. As described in this article, at 16–17 days of age, two groups of chickens of similar body weights were constituted (N = 7 per group). The fed immuno-neutralized group received three i.v. injections of anti-porcine insulin guinea pig serum (1.5 ml/kg) (PromoCell, Heidelberg, Germany) at times 0, 2 and 4 h. Guinea pig serum was used as the vehicle for the anti-porcine insulin. The fed control group received three i.v. injections delivering only normal guinea pig serum (1.5 ml/kg) at times 0, 2 and 4 h. At 5h post-injection, chickens were slaughtered and 2 different types of muscle, a fast-twitch glycolytic muscle (*Pectoralis major*) and a mixed type muscle (leg muscle) were sampled, snap frozen in liquid nitrogen and stored at -80°C until analysis.

### GLUT12 translocation experiment

Male broiler chickens were fasted overnight or fed and injected with insulin (1U/kg) by intraperitoneal injection or not injected (N = 3 per group). Broiler chickens were slaughtered one hour after insulin injection and leg muscles were sampled and snap frozen in liquid nitrogen.

### RNA isolation and RT-PCR

Total RNA was extracted from 100 mg tissue samples (200 mg for adipose tissue samples) using RNA Now (Biogentec, Seabrook, TX, USA) according to the manufacturer’s recommendations. After RNase-Free DNase treatment (Ambion, Clinisciences, Montrouge, France), RNA was reverse-transcribed using Super Script II RNase H Reverse Transcriptase (Invitrogen, Carlsbad, CA, USA) with Random Primers (Promega, Charbonnieres-les-Bains, France). The specifically designed and validated sequences of primers used for identification of chicken *SLC2A12* were: (Forward) 5’ AGA-GAG-TGG-GGA-GGT-TCC-C 3’; (Reverse) 5’ TCA-GGA-CGA-GCC-AAG-ACA 3’. *SLC2A12* mRNA was detected by reverse transcriptase-polymerase chain reaction (RT-PCR) in various chicken tissues.

cDNA samples were subsequently amplified in triplicate by real time PCR using Sybr Green I Master kit (Roche, Mannheim, Germany) and a LighterCycler® 480 II apparatus (Roche, Meylan, France). β-actin and cytochrome (Cyt b) messengers were measured as references using primers previously validated for β-actin [14, 26] or primers specifically designed and validated for chicken cytochrome b (Cyt b) ((Forward) 5’ CGG- ACG-AGG-CCT-ATA-CTA-CG 3’; (Reverse) 5’ GGG-AGA-ACA-TAG-CCC-ACA-AA 3’). Gene expression levels were estimated on the basis of PCR efficiency and threshold cycle (Ct) deviation of an unknown sample versus a control, as previously described [27].

### Western blotting

Tissue lysates were prepared as previously described [28] to analyse the tissue distribution of GLUT12. Lysates were centrifuged at 1000 g for 30 min at 10°C and supernatants were then centrifuged at 31000 g for 45 min at 10°C. Solubilized lysates (80 μg of protein) were subjected to SDS-PAGE and Western blotting using anti-GLUT12, then anti-vinculin antibodies (after stripping). GLUT12 is a polyclonal antibody. After washing, membranes were incubated with a DyLight® 680 fluorescent antibody. Bands were visualized by Infrared Fluorescence using the Odyssey® Imaging System (LI-COR Inc. Biotechnology, Lincoln, NE) and quantified by Odyssey infrared imaging system software (Application software, version 1.2).

For GLUT12 translocation studies, membrane and cytosolic proteins were extracted with the Promokine commercial kit (Promocell) according to the manufacturer’s recommendations using 750 mg muscle samples. The plasma membrane proteins extraction procedure using this commercial kit has been validated through different publications in the literature [29, 30] and in chickens using antibodies directed against markers specific of plasma membranes (i.e NaK ATPase and CD56). Moreover, we checked NaK ATPase enrichment in the membrane after insulin stimulation [31]. GLUT12 was detected and quantified as above.

### Statistical analysis

Values are presented as means ± SEM. Data were processed using the Statview Software program, version 5 (SAS Institute, Cary, NC, USA). Data were subjected to analysis of variance (ANOVA) to detect significant intergroup differences (Tissue comparison; Fed vs Ins immunoneutralised). The means were compared by Fisher’s least significant difference test in the case of a significant effect. P<0.05 was considered statistically significant.

## Results

### Further evidence of the absence of GLUT4 in the chicken genome

**Fig 1:**
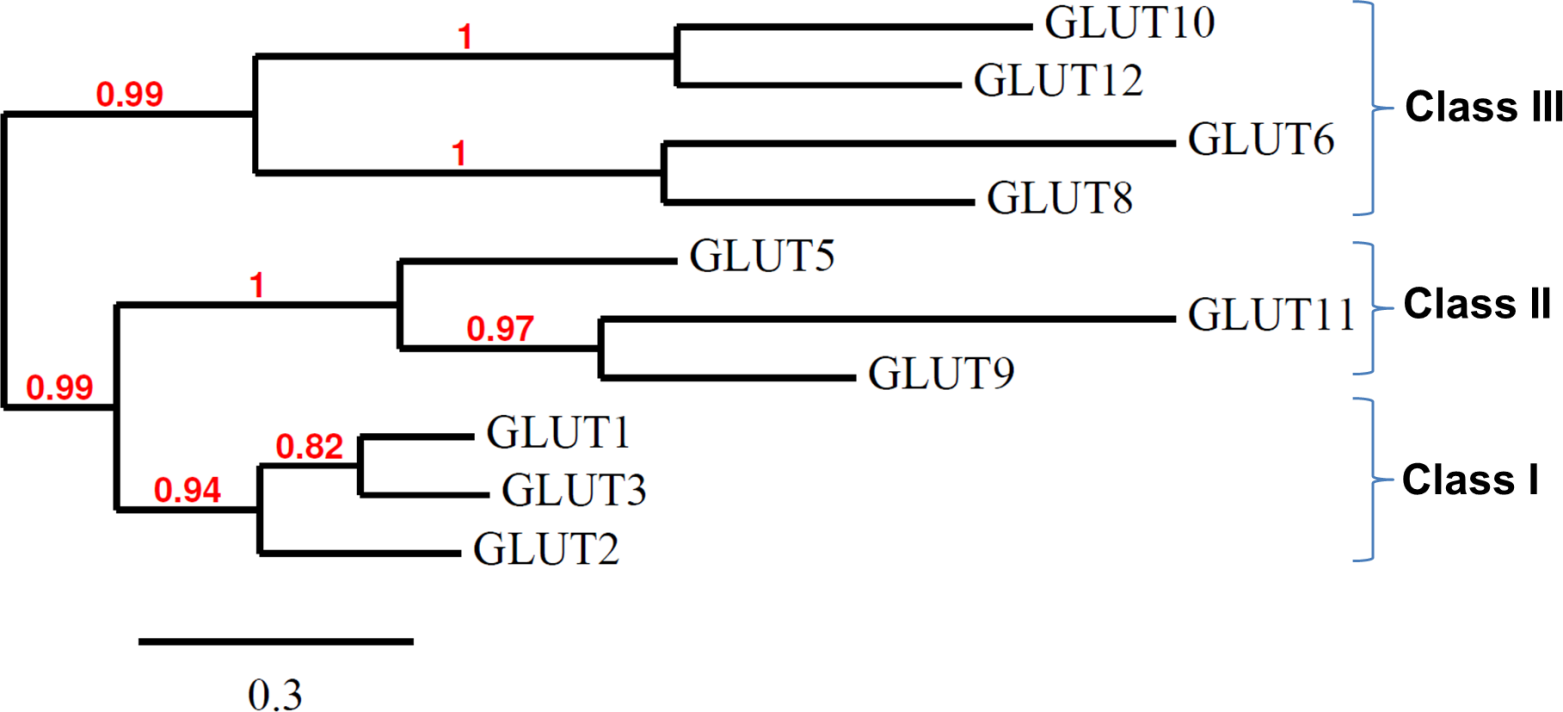
Phylogenetic tree of multiple alignments of all known chicken GLUT protein sequences. The analysis of chicken GLUTs was performed on the Phylogeny.fr platform (http://www.phylogeny.fr; [20, 21]) using Ensembl protein IDs. The tree was constructed as described in Materials and Methods. Reliability of internal branches was assessed using the aLRT test. The scale represents the substitution rate.

Up to ten predicted and annoted GLUTs were found in the Ensembl chicken database (Ensembl release 75; Table 1). According to the phylogenetic analysis of these chicken GLUTs (Fig 1), we discriminated three main branches corresponding to the three classes of GLUTs described in mammals: Class I (GLUT1, 2, and 3), Class II (GLUT5, 9 and 11) and Class III (GLUT6, 8, 10 and 12). The different chicken GLUTs showed 24 to 69% of identity between the different paralogs, highest percentage of identity being found between GLUT1 and GLUT3 (Table 3).

**Table 3:** Maximum identity between the different chicken GLUT proteins.

GLUTs	GLUT1	GLUT2	GLUT3	GLUT5	GLUT6	GLUT8	GLUT9	GLUT10	GLUT11	GLUT12
**GLUT1**	**100**	55 _(95)_	69_(100)_	44 _(90)_	28 _(94)_	30 _(94)_	40_(98)_	33 _(81)_	37_(97)_	30_(78)_
**GLUT2**	55^δ^_(95)*_	**100**	52_(95)_	37 _(96)_	30 _(80)_	29 _(83)_	33 _(96)_	32 _(78)_	32 _(97)_	29 _(74)_
**GLUT3**	69_(100)_	52_(95)_	**100**	41_(96)_	28_(91)_	30 _(94)_	40_(96)_	31_(89)_	34 _(93)_	32_(90)_
**GLUT5**	44 _(90)_	37 _(96)_	41_(96)_	**100**	28 _(79)_	31 _(84)_	46 _(97)_	27 _(76)_	42 _(93)_	26 _(69)_
**GLUT6**	28 _(94)_	30 _(80)_	28_(91)_	28 _(79)_	**100**	45 _(95)_	26 _(81)_	33 _(80)_	25_(94)_	24 _(83)_
**GLUT8**	30 _(94)_	29 _(83)_	30 _(94)_	31 _(84)_	45 _(95)_	**100**	27 _(84)_	37 _(86)_	26 _(80)_	28_(94)_
**GLUT9**	40 _(98)_	33 _(96)_	40_(96)_	46 _(97)_	26 _(81)_	27 _(84)_	**100**	25 _(74)_	42 _(97)_	27 _(81)_
**GLUT10**	33 _(81)_	32 _(78)_	31_(89)_	27 _(76)_	33 _(80)_	37 _(86)_	25 _(74)_	**100**	27 _(90)_	42_(95)_
**GLUT11**	37_(97)_	32 _(97)_	34_(93)_	42 _(93)_	25_(94)_	26 _(80)_	42 _(97)_	27 _(90)_	**100**	25 _(73)_
**GLUT12**	30_(78)_	29 _(74)_	32_(90)_	26 _(69)_	24 _(83)_	28_(94)_	27 _(81)_	42_(95)_	25 _(73)_	**100**

^δ^: % of identity

_(x)*:_ Query coverage (%)

**Fig 2:**
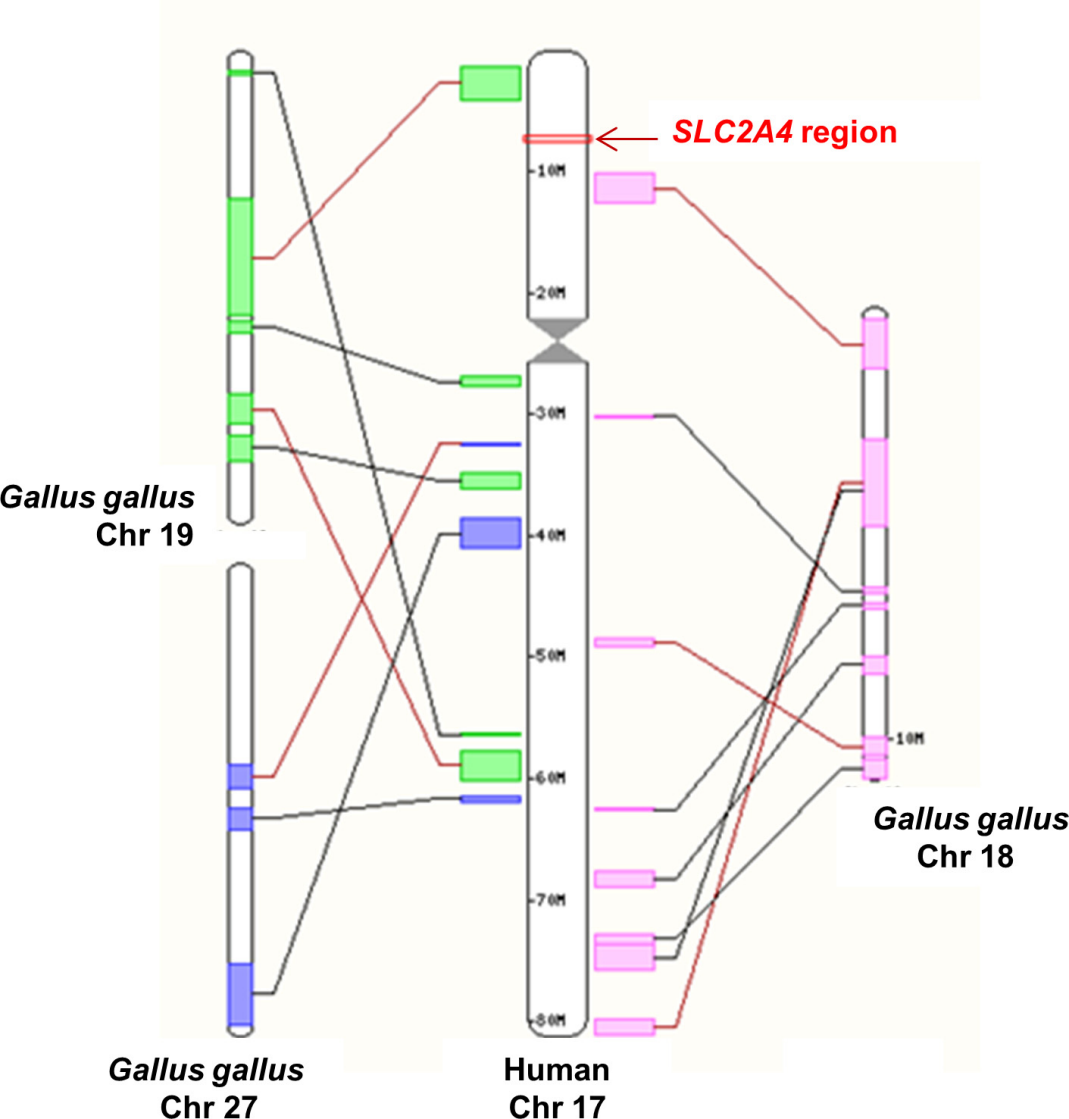
Comparative maps of *SLC2A4* genomic region. Synteny blocks between human Chromosome 17 (Chr 17) and chicken chromosomes 18, 19 and 27 (Chr 18, 19 and 27) according to Ensembl (http://www.ensembl.org). The arrow indicates the *SLC2A4* region in human Chr17.

The chicken genome sequencing available to date has not provided any evidence regarding the presence of a chicken GLUT4 although a GLUT4 ortholog or *SLC2A4* gene has been found in various species of fish (Cod, Stickleback, Fugu, Tetraedon, Platyfish, Medaka and Tilapia), as well as in Sauropsida (Chinese softshell turtle) and Reptilia (Anole lizard) classes (data not shown). No sequence encoding a *SLC2A4* ortholog has been found in other birds (Zebra finch, Turkey or Duck), the Frog or Zebrafish. The human *SLC2A4* gene is located on chromosome 17 in a synteny block that is well conserved within vertebrates (data not shown). The region in which the human *SLC2A4* gene is located is almost entirely missing from the current chicken sequence assembly (Fig 2).

### Is there another insulin-sensitive GLUT in the chicken?

#### Phylogenetic analysis of GLUT12

**Fig 3:**
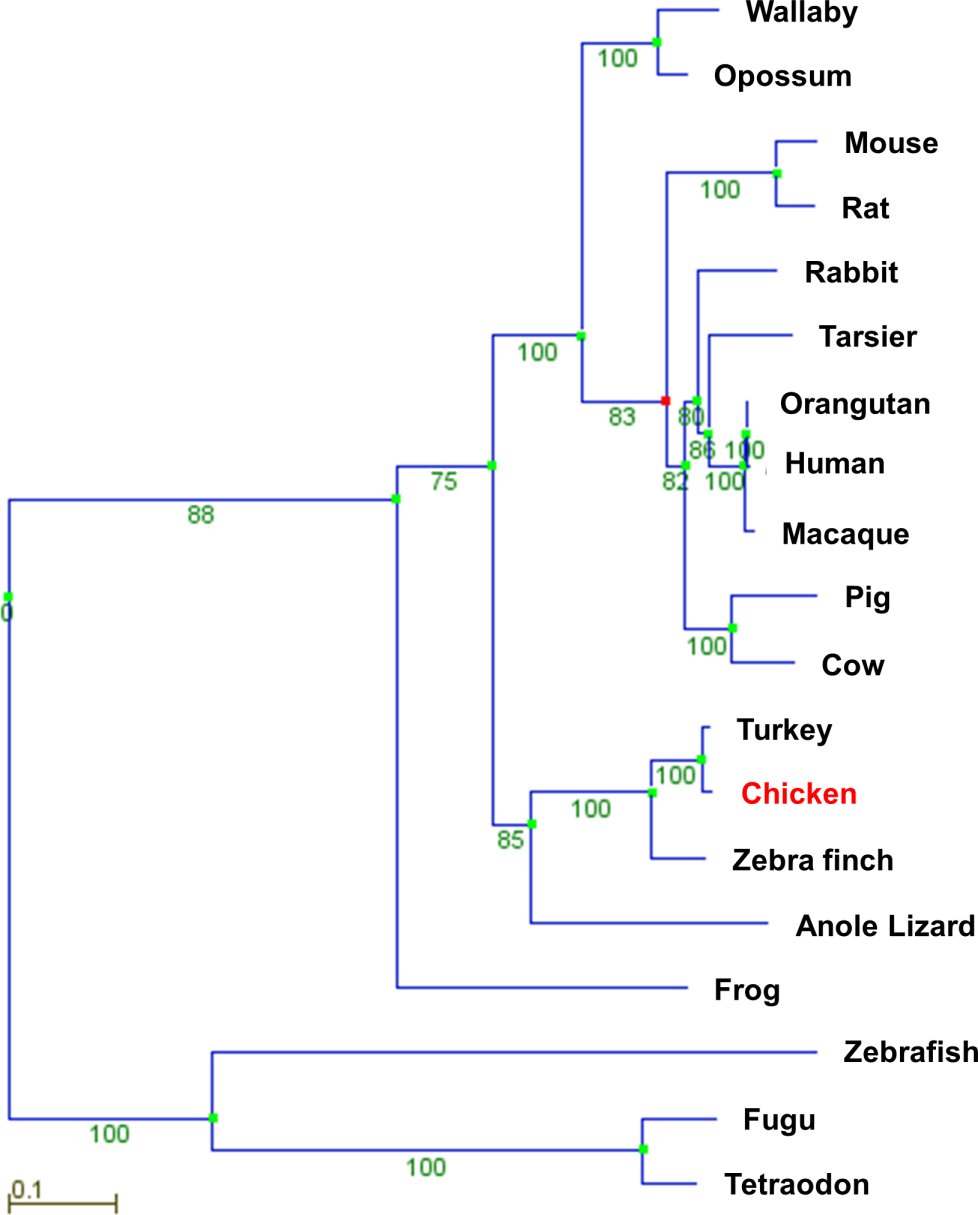
Phylogenetic tree of protein sequence of chicken GLUT12 and GLUT12 from other vertebrates (see Table 2). The tree was constructed as described in Materials and Methods using PhyleasProg analysis web server (Phylogenetic Analysis Programs v2.7).

**Fig 4:**
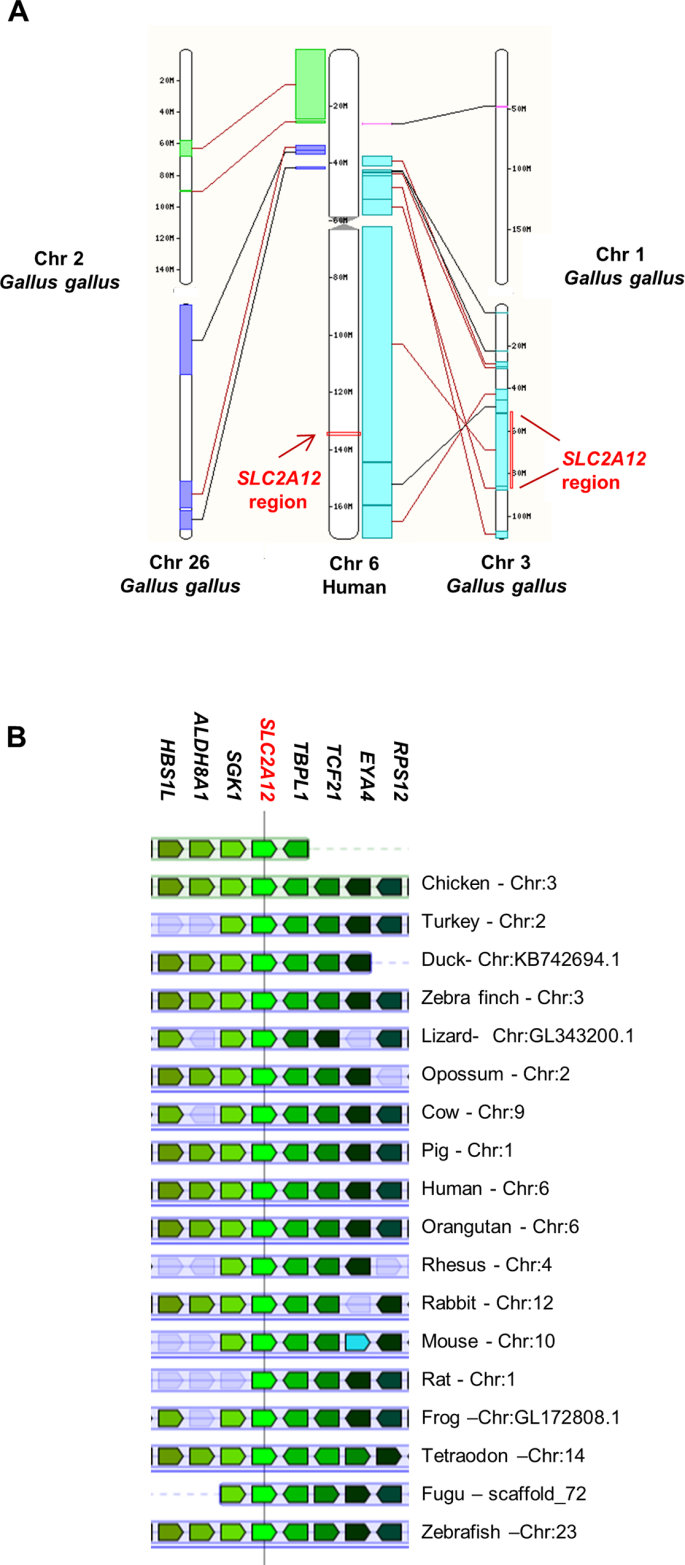
Comparative maps of *SLC2A12* genomic region. (A) The genomic location of *SLC2A12*. synteny blocks between human Chromosome 6 (Chr 6) and chicken chromosomes 1, 2, 3 and 26 (Chr 1, Chr 2, Chr3 and Chr 26) according to Ensembl (http://www.ensembl.org). The arrows indicate *SLC2A12* regions. (B) Gene arrangement of the genomic region encompassing *SLC2A12* according to the genome browser Genomicus (http://www.genomicus.biologie.ens.fr, Genomicus—database version: 79.01 / Web-code version: 2014-09-19). Schematic gene maps of the conserved syntenic regions of *SLC2A12* in *Gallus gallus* and in other vertebrates.The gene initially placed in the centre of the display and aligned over a vertical black line is the gene that was used as query (reference gene = *SLC2A12*). Coloured genes over a light blue background corresponds to extant or ancestral genes that are orthologous to genes from the species used in the query that show the same colour. The coding direction of the genes is indicated by the pointed end. Chr: denotes the chromosome. Neighbouring genes upstream and downstream in the chicken and other species: *SGK1*, serum/glucocorticoid regulated kinase 1; *ALDH8A1*, aldehyde dehydrogenase 8 family, member A1; *HBS1L*, uncharacterized protein; *TBPL1*, TBP-like 1; *TCF21*, transcription factor 21; *EYA4*, eyes absent homolog 4; *RPS12*, ribosomal protein S12.

The analysis of chicken GLUTs (Table 1) showed the existence of a GLUT12 ortholog (corresponding to the *SLC2A12* gene) in the chicken. The *SLC2A12* gene is located on chromosome 3, and comprises 5 exons and encodes a putative 596 amino acid protein. The phylogenetic analysis of GLUT12 amino acid sequences in different species (Table 2) is presented in Fig 3. We decided to determine whether selection pressures acted on the avian GLUT12 sequence. No positive selection pressure was evidenced in these 19 species. Synteny analysis and comprehensive genome location searches (Fig 4A and 4B) showed a similar arrangement of the *SLC2A12* gene and its neighbouring genes upstream and downstream in the chicken and other species.

#### Sequence analysis of avian GLUT12 protein

**Fig 5:**
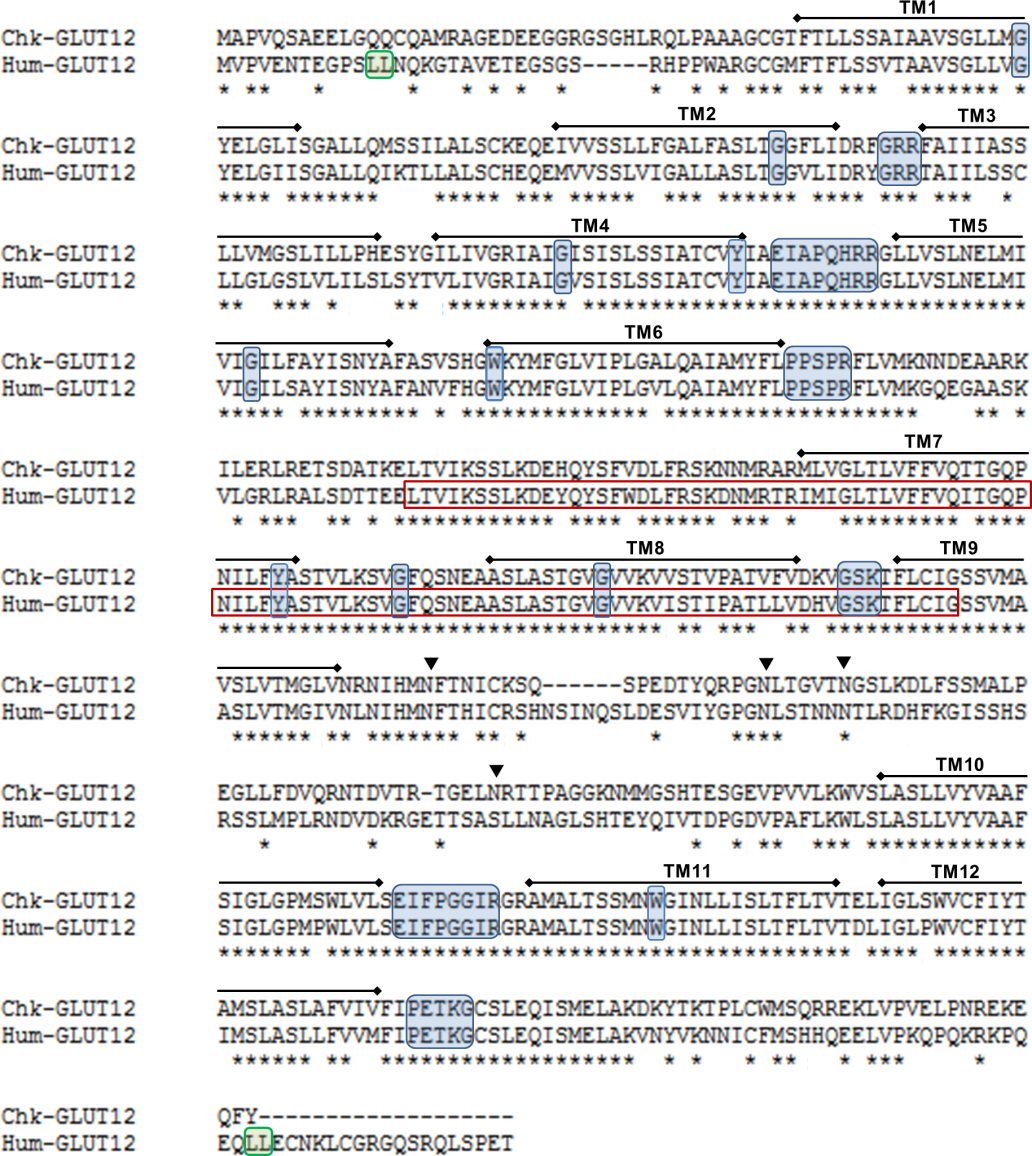
Amino acid alignment of chicken GLUT12 amino acid sequence with human GLUT12 sequence. Alignment was performed using Clustalw algorithm. Amino acids are represented by the single letter code. Gaps in the amino acid sequences are indicated with a dash (-).* indicates positions which have a single, fully conserved residue. Lines with numbers indicate the 12 transmembrane domains (TM). (▼) indicates the predicted N-glycosylation sites. Other important conserved residues and motifs such as those corresponding to specific sugar transporter signatures are highlighted in blue boxes. NH2 and COOH terminal dileucine motifs are highlighted in green boxes. In the human amino acid sequence, the red box corresponds to the human peptide used to produce the anti-GLUT12 antibody.

The chicken *SLC2A12* gene encodes a protein which exhibits 71% sequence identity to human GLUT12. Alignment of the deduced amino acid sequence of chicken and human GLUT12 proteins showed that chicken GLUT12 possesses all the features essential for sugar transport: 12 membrane-spanning helices, intracellular NH2 and COOH termini and conserved amino acid residues and motifs important for sugar transport activity (i.e., seven glycine residues, tryptophan in helices 6 and 11, tyrosine in helices 4 and 7) (Fig 5).The amino acid sequence of GLUT12 presented at least four putative extracellular N-linked glycosylation sites in the larger loop 9 at amino acid residues 376, 395, 401 and 434, as predicted using the GlycoEP webserver [32]. The di-leucine motifs (LL) present at the NH2 and COOH termini of human GLUT12, which are considered to mediate internalization, were not found in chicken GLUT12 (Fig 5).

#### Tissue expression of SLC2A12 mRNA and protein distribution of GLUT12 in chicken tissues

**Fig 6:**
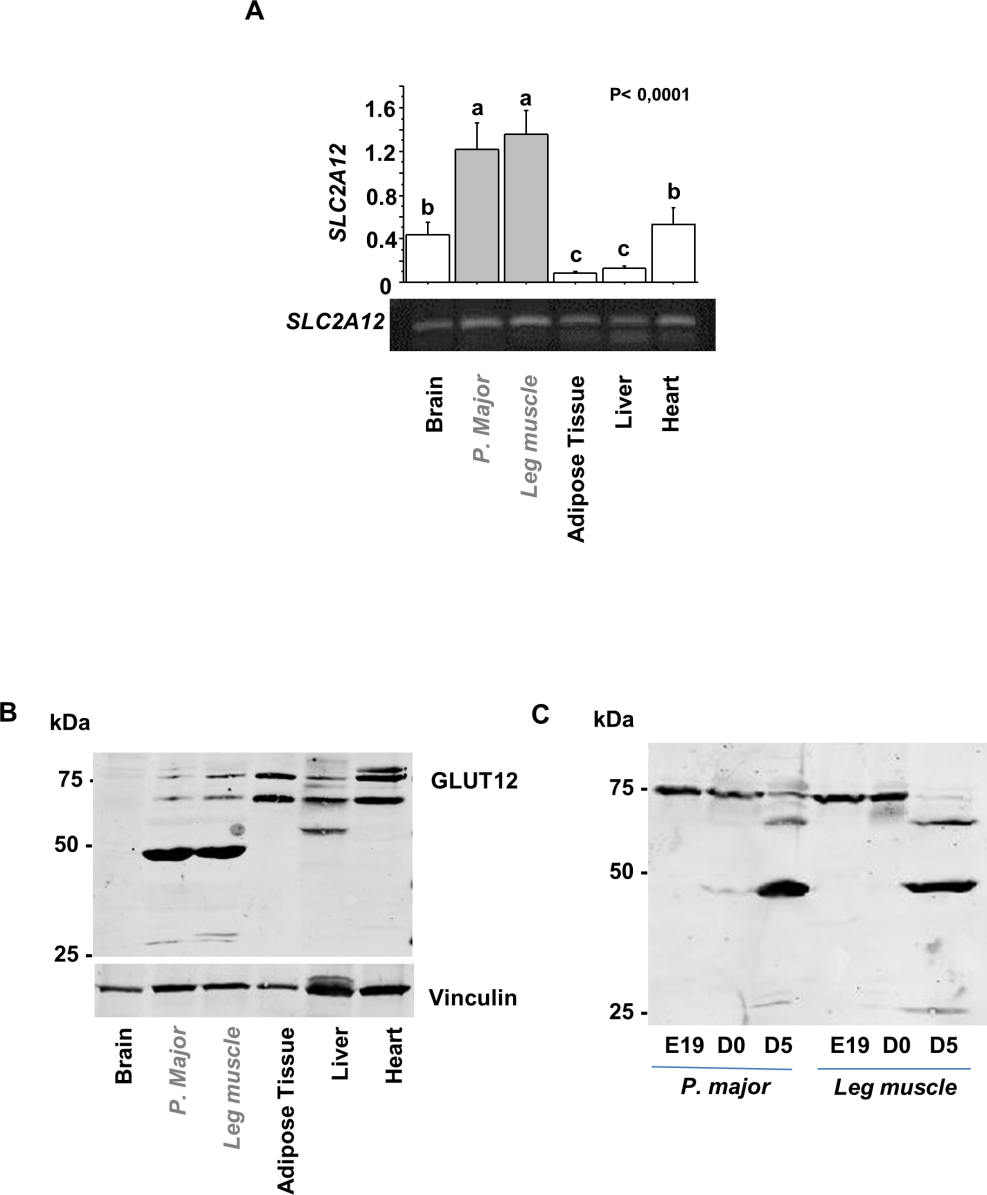
Tissue expression pattern of GLUT12 in the chicken. (A) Tissues from fed chickens were analysed for *SLC12A12* expression by Real-Time PCR (N = 6). Values represent means and standard errors. Data were subjected to analysis of variance (ANOVA) to detect significant intergroup differences. Statistical model was tissue comparison. The means were compared by Fisher’s least significant difference test. P<0.05 was considered statistically significant. (B) Tissues from fed chickens were analysed by Western blotting using an anti-GLUT12 antibody for protein distribution analyses (N = 5). In grey: skeletal muscles (*Pectoralis major* and leg muscle). Vinculin was used as a protein loading control. (C) Protein distribution was analysed from *Pectoralis major* and the leg muscles, collected at 19 days during embryogenesis, hatch and 5 days post-hatch. (N = 7).

The tissue expression pattern of the *SLC2A12* messenger RNA was analysed in various tissues from fed chickens by real-time PCR. *SLC2A12* mRNA was detected in all tissue samples, but was the most abundant in skeletal (*Pectoralis major* and leg muscles) muscles, i.e. insulin-sensitive tissues (Fig 6A).

Tissue distribution of GLUT12 protein was analysed by Western blotting using an antibody against human GLUT12 (Fig 6B). Immuno-reactive bands around the expected size (75 kDa) were recognized in insulin-sensitive tissues such as skeletal muscles, adipose tissue and heart. Another signal, that was stronger than that at 75 kDa, was also detected at around 50 kDa in skeletal muscle lysates but not in other tissues. This band at 50 kDa is detected only in chicken muscle lysates from animals after hatch and not in muscles lysates from embryo (E19) (Fig 6C). At E19, the 75 kDa form was highly detected and then decreased strongly at hatch. Conversely, the 50 kDa form appeared at hatch and then increased strongly at Day 5 and is still detected at 6 weeks of age.

#### Insulin-sensitivity of chicken SLC2A12 /GLUT12

**Fig 7:**
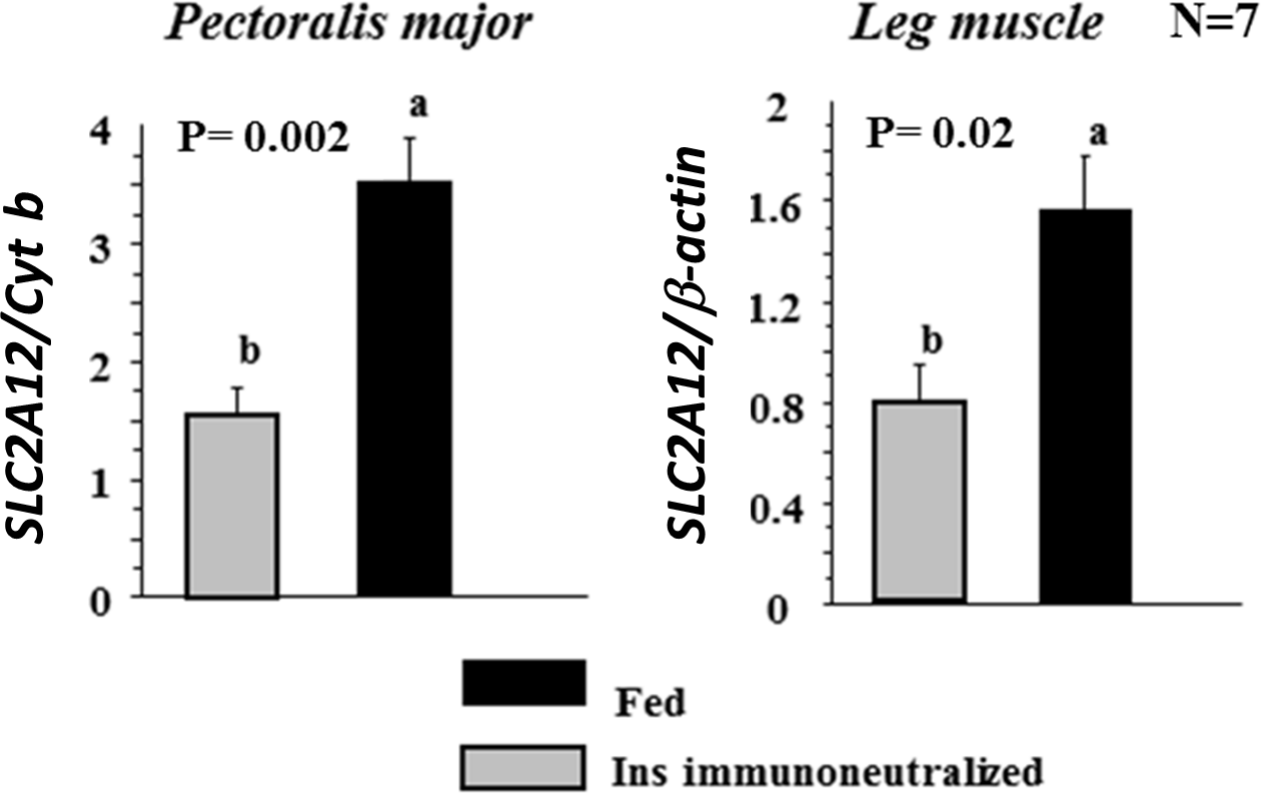
Effects of insulin on *SLC2A12* gene expression in skeletal muscles. mRNA levels of *SLC2A12* were measured by Real-Time PCR in *Pectoralis major* and leg muscles of fed chicken controls (Fed group) and fed chickens injected with an anti-insulin antibody (Ins immuno-neutalized group) (N = 7). Cytochrome b (Cytb) and β-actin were used as housekeeping genes.Values represent means and standard errors. Data were subjected to analysis of variance (ANOVA) to detect significant intergroup differences (Fed vs Ins immuno-neutralized). The means were compared by Fisher’s least significant difference test. P<0.05 was considered statistically significant.

We next analysed the effects of insulin on *SLC2A12* gene expression in skeletal muscles using an insulin immuno-neutralization model previously described and characterized [14]. *SLC2A12* gene expression was measured 5h post-injection by qRT-PCR in two different types of muscle: a glycolytic muscle (*Pectoralis major*) and a mixed type muscle (leg muscle). *SLC2A12* mRNA expression was significantly lower in the insulin-immuno-neutralized condition compared to controls in both muscle types (*Pectoralis major* and leg muscles) (Fig 7).

The last issue addressed in the present study was the existence of potential regulation of GLUT12 at the protein level. More extreme experimental models were used: fasted vs. fed states and acute insulin injection (1U/kg).

**Fig 8:**
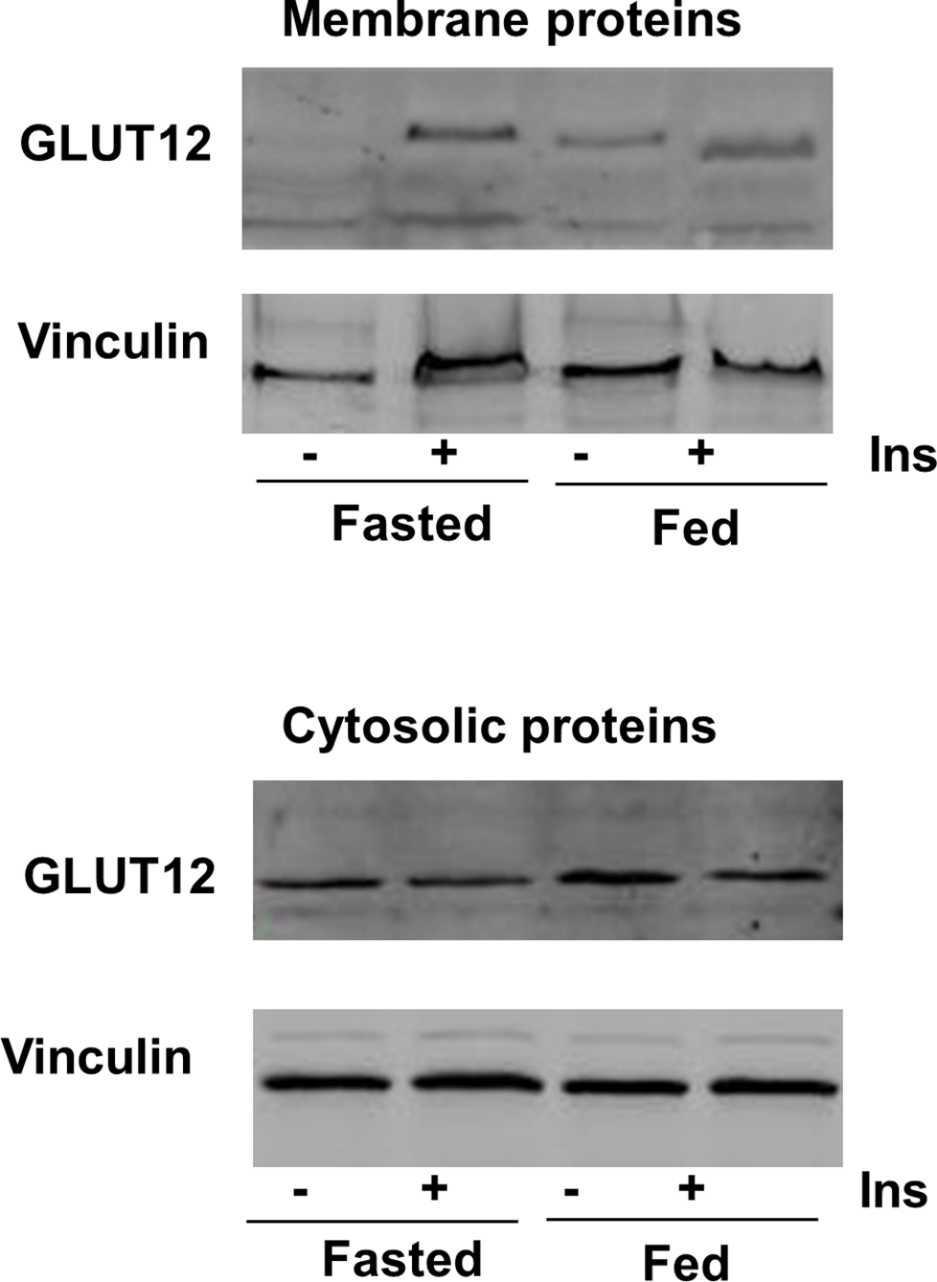
GLUT12 translocation after insulin stimulation. In the upper part, a representative Western blot shows GLUT12 content in membrane fractions prepared from leg muscle of fasted and fed animals without insulin injection or 1hr after insulin injection (1U/kg). In the lower part, a representative Western blot shows GLUT12 content in the corresponding cytosolic proteins used as a control of the cytosolic GLUT12 content. Vinculin was used as a loading control for the two fractions.

Membrane and cytosolic proteins were prepared from leg muscles and analysed by Western blotting (Fig 8). The 75 kDa immuno-reactive band was observed in membrane fractions (Fig 8, upper part) from fed animals in the absence of insulin injection, but not in fasted animals. Membrane GLUT12 protein significantly increased in fasted and fed conditions in response to insulin injection. In the cytosolic fraction (Fig 8, lower part), the immuno-reactive GLUT12 band was detected at identical levels, whatever the experimental treatment. These observations demonstrate that GLUT12 translocates to the plasma membrane in response to insulin in chicken skeletal muscle.

## Discussion

In mammals, GLUT4 is responsible for glucose uptake by insulin-sensitive tissues such as skeletal muscle, heart and adipose tissue. Up to now, the chicken genome database contains several sequences that are suggested as encoding glucose transporter-like proteins, but no one encoding a GLUT4 ortholog. Phylogeny and synteny analyses confirmed the lack of GLUT4 in chickens although a GLUT4 ortholog or *SLC2A4* gene has been found in various species of fish, as well as in Sauropsida (Chinese softshell turtle) and Reptilia (Anole lizard) classes. Moreover, the region in which the human *SLC2A4* gene is located is almost entirely missing from the current chicken sequence assembly, suggesting that GLUT4 may have been lost in bird lineages. The absence of GLUT4 in chicken could explain partially the particular features of glucose metabolism exhibited by the avian species. Nevertheless chickens are not totally insensitive to exogenous insulin and the existence of a functional insulin-sensitive glucose transport in avian muscle has been demonstrated even though it remains lower compared to mammals [12, 14–16]. Identity of the GLUT involved is controversial. In one study conducted on ducks, an immuno-reactive band has been detected at the expected size for GLUT4 using an anti-rat GLUT4 antibody and shown to translocate to the plasma membrane in response to insulin administration and in parallel to glucose use by the leg [13]. Nevertheless, this band has never been sequenced to confirm that it is really a GLUT4 ortholog and not another GLUT showing a high sequence homology. Moreover, no sequence encoding a *SLC2A4* ortholog has been found in ducks. GLUT1 has also been suggested, but no change in GLUT1 quantity was shown using membranes prepared from myotubes incubated under conditions where glucose transport was increased [15].

Mice that have a complete ablation of GLUT4 are growth retarded and have a significant cardiac hyperthophy, reduced body weight and adiposity levels and are less sensitive to insulin [33]. Whereas specific deletion of muscle GLUT4 does not affect glucose disposal and glucose tolerance suggesting that compensation from the transporters may contribute to this unaltered homeostasis of glucose and that another GLUT may also be involved in the regulation of whole body glucose homeostasis [34]. Recent findings suggest that GLUT12 might contribute to insulin-stimulated glucose uptake in insulin-sensitive tissues, in addition to GLUT4 [4–7]. Indeed, transgenic overexpression of GLUT12 in mice enhances insulin sensitivity with no change in GLUT4 content [6] and a compensatory increase in the expression of GLUT12 was observed in genetically altered mice lacking GLUT4 [35]. At the protein level, GLUT12 was also significantly elevated in the myocardium of GLUT4 null mice compared to wild type controls [36]. A sequence encodes a chicken GLUT12 ortholog. The phylogenetic analyses demonstrated that no specific changes in *SLC2A12* gene occurred during evolution and thus strongly supported a similar evolution of the *SLC2A12* gene in avian species and other vertebrates, suggesting a potential similar role of GLUT12 in avian species and other vertebrates. As described in mammals [4], GLUT12 messenger is expressed in insulin-sensitive tissues, such as skeletal muscles, the heart and adipose tissue in the chicken. The GLUT12 expression was insulin-dependent in the muscles investigated (*Pectoralis major* or leg muscle). Interestingly, a similar result has been described in zebrafish [37], a species lacking GLUT4 [38]. GLUT12 protein is expressed in the skeletal muscles (*Pectoralis major* and leg muscles), heart and adipose tissue under different forms: two high molecular forms at around 75kDa (particularly clear in the heart and the adipose tissue), the expected size according to the amino acid sequence, and a low molecular form at 50 kDa exclusively in skeletal muscles. The different bands detected around 75 kDa could correspond to different glycosylated forms as suggested by the four putative extracellular N-linked glycosylation sites and preliminary deglycosylation experiments (S1 Fig). These findings are however still to be clearly demonstrated. The higher bands around 75 kDa form are enriched at the cell membrane of fed chickens when plasma insulin levels are typically high or under insulin stimulation (compared to fasted chickens). Therefore insulin administered *in vivo* stimulates the translocation of GLUT12 to the plasma membrane in chicken skeletal muscles. In mammals, GLUT12 translocation seems to depend on the tissues and/or the presence of GLUT4. Indeed, there is still some controversy whether in the heart GLUT12 might be regulated by insulin [36]. In a healthy myocardium, insulin stimulation did not increase translocation of GLUT12, whereas it increased translocation of GLUT4. Cell membrane GLUT12 content was only increased in the diabetic myocardium, potentially as a compensatory mechanism for the observed down regulation of GLUT4.

Some study described the importance of post-translational modifications of GLUT4 for its subcellular location and translocation [39]. Among potential post-translational modifications, a conserved N-glycosylation consensus site in GLUT4 is positioned in the first extracellular loop. Critical roles for this N-glycan chain in intracellular trafficking and stability of GLUT4 have been shown and confirmed using an inhibitor of endoplasmic reticulum (ER) / Golgi α-mannosidase I (kifunensine, KIF) or a GLUT4 mutant lacking the N-glycosylation site [39–41]. The amino acid sequence of chicken GLUT12 presented at least four putative extracellular N-linked glycosylation sites in the larger loop 9. These N-glycosylation sites are a characteristic of Class III facilitated glucose transporters. A similar N-linked glycosylation motif was found in K^+^Cl^-^ cotransporters (KCCs) of the SLC12 family, and site-directed mutagenesis approaches have demonstrated that its glycosylation is essential for regulating cell surface expression, stability and activity of KCC4 proteins [42].

The GLUT12 form of lower molecular weight (50 kDa) was exclusively detected in chicken skeletal muscles after hatch. Similar signals at around 50 kDa have been reported for GLUT12 in bovines and goats [43–44]. A strong resistance to the effect of insulin on glucose metabolism has been reported in ruminant animals [45]. The authors suggest that the low sensitivity to insulin may be related to the cytoplasmic distribution of ruminant GLUT12 lacking the dileucine motif (LL) [43–44]. The dileucine motif typical for internalization has been described in human GLUT12 sequence but is not retrieved in chicken GLUT12 nor in species considered as insulin-resistant such as goat or bovine [43–44]. This di-leucine motif in C-term has been also described for another insulin-sensitive transporter GLUT4 and it was not retrieved in fish GLUT4 sequence [46]. Despite the lack of this motif and differences in the GLUT4 traffic characteristics, fish glucose transporter GLUT4 can translocate to the cell surface in response to insulin in skeletal muscle cells. The chicken GLUT12 as well as the fish GLUT4 presented some particular features that could result in differences in protein trafficking and stability on the cell surface and might partially explain their atypical glucose metabolism and their natural insulin resistance [47–48]. We are conscious that an extensive characterization of their traffic is now required. Its purification and characterization are necessary to better know the nature of this form and determine if the detected signal corresponds to an immature form or a truncated form of GLUT12 and its role in glucose metabolism.

In the chicken, therefore, GLUT12 may act as an insulin-sensitive transporter because it is expressed in insulin-sensitive tissues and it can be recruited to the plasma membranes in fed conditions or following insulin injection. Nevertheless, despite considerable similarity of protein sequence with mammalian GLUT12, the chicken GLUT12 presented some particular features that could result in differences in protein trafficking and stability on the cell surface membrane in the chicken. The consequences might partially explain the low sensitivity of chickens to exogenous insulin. Extensive characterization of the GLUT12 trafficking as well as its regulation in the chicken is now required.

## Supporting Information

S1 Fig Deglycosylation of GLUT12 from chicken leg muscle and adipose tissue. Leg muscle and adipose tissue lysates were prepared using Enzymatic DeGlycoMx Kit from QA bio and incubated for 3 hours at 37°C according to the manufacturer’s recommendations to analyse the glycosylations of GLUT12. Representative immunoblot of no deglycosylated samples (Ctl) and deglycosylated samples (DeGly: deglycosylated). Membrane was probed with the anti-GLUT12 antibody, and then anti-vinculin antibodies (after stripping).
(PDF)
