# Supplementary figures and images for: Phylogenesis and Biological Characterization of a New Glucose Transporter in the Chicken (Gallus gallus), GLUT12
Source: PLoS One. 2015 Oct 2;10(10):e0139517. doi: 10.1371/journal.pone.0139517 (PMC4592010; doi:10.1371/journal.pone.0139517)

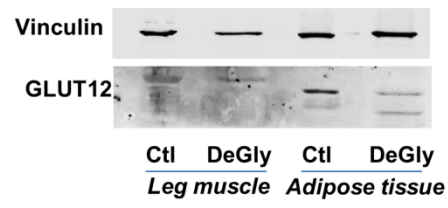

Supplement: S1 Fig — Leg muscle and adipose tissue lysates were prepared using Enzymatic DeGlycoMx Kit from QA bio and incubated for 3 hours at 37°C according to the manufacturer’s recommendations to analyse the glycosylations of GLUT12. Representative immunoblot of no deglycosylated samples (Ctl) and deglycosylated samples (DeGly: deglycosylated). Membrane was probed with the anti-GLUT12 antibody, and then anti-vinculin antibodies (after stripping). (PDF) [file pone.0139517.s001.pdf]
